# Supplementary material for: Accuracy and Clinical Relevance of Intra-Tumoral Fusobacterium nucleatum Detection in Formalin-Fixed Paraffin-Embedded (FFPE) Tissue by Droplet Digital PCR (ddPCR) in Colorectal Cancer
Source: Diagnostics (Basel). 2022 Jan 5;12(1):114. doi: 10.3390/diagnostics12010114 (PMC8775036; doi:10.3390/diagnostics12010114)
Supplement: Supplementary file 1 [file diagnostics-12-00114-s001.zip › diagnostics-1410421-supplementary.pdf]

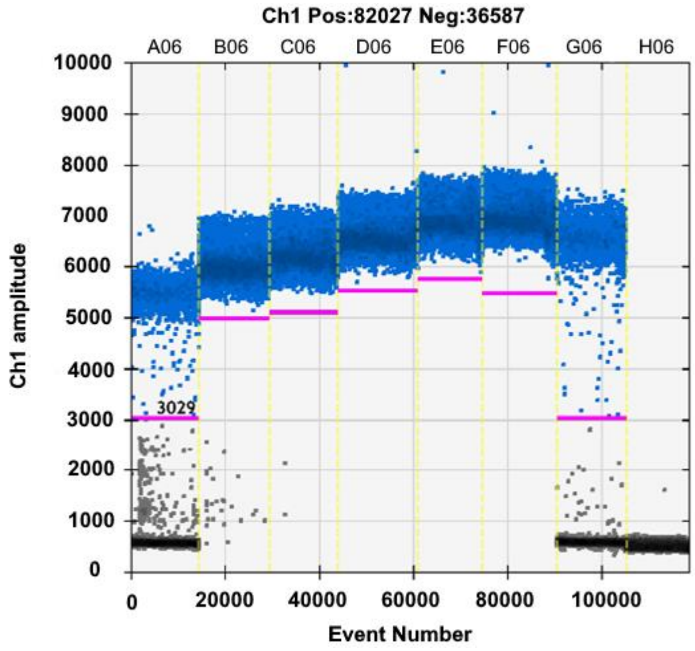

Supplementary Figure S1: One-dimensional graph generated by the QuantaSoft software (BioRad) showing the temperature gradient test. A06: temperature of 63.8°C; B06: 63.2°C; C06: 61.9°C; D06: 60°C; E06: 57.8°C; F06: 55.9°C; G06: 54.6°C and H06: No template control (NTC).

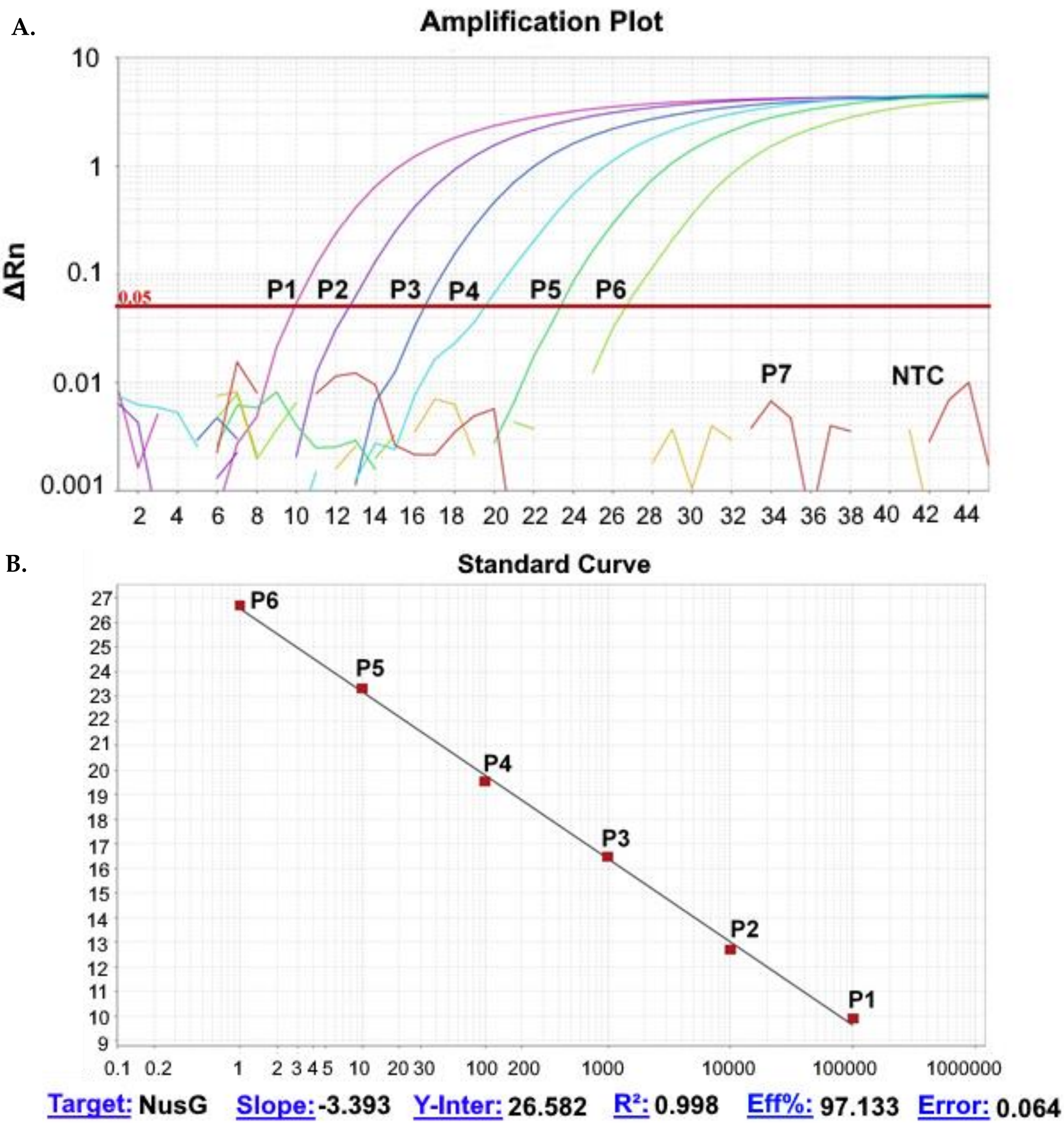

Supplementary Figure S2: Amplification (A) and standard curves (B); generated from qPCR results of the detection of Fn DNA in serial dilutions of *Fusobacterium nucleatum* DNA and *E. coli* DNA confirming the efficiency of the primers and probe, according to the values presented in the correlation coefficient (R^2) and efficiency,

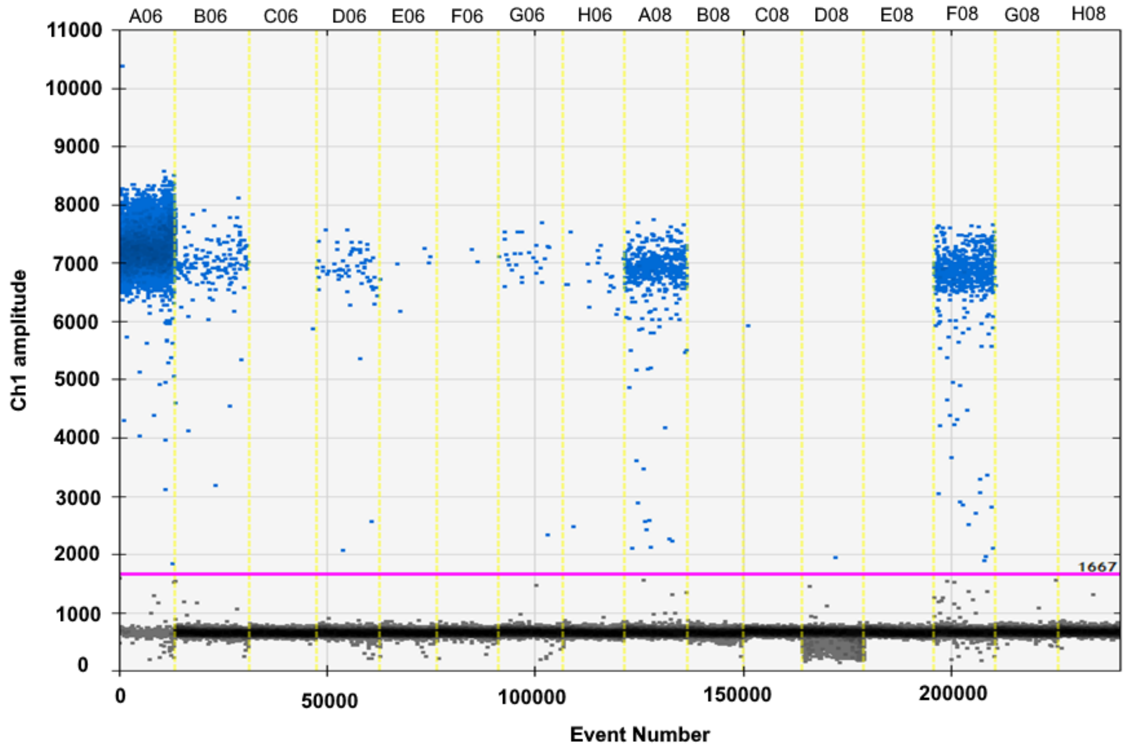

Supplementary Figure S3: One-dimensional graphics generated by the QuantaSoft software (BioRad) showing the detection of *Fn* DNA in FFPE tumor tissue assay of patients with CRC. A06: *Fusobacterium nucleatum* DNA (positive control); B06, D06, E06,F06, G06, H06, A08 and F08: *Fn* positive samples; C06, B08, C08, D08 and E08:*Fn* negative samples; G08: *E. coli* DNA (negative control) and H08: No template control (NTC).

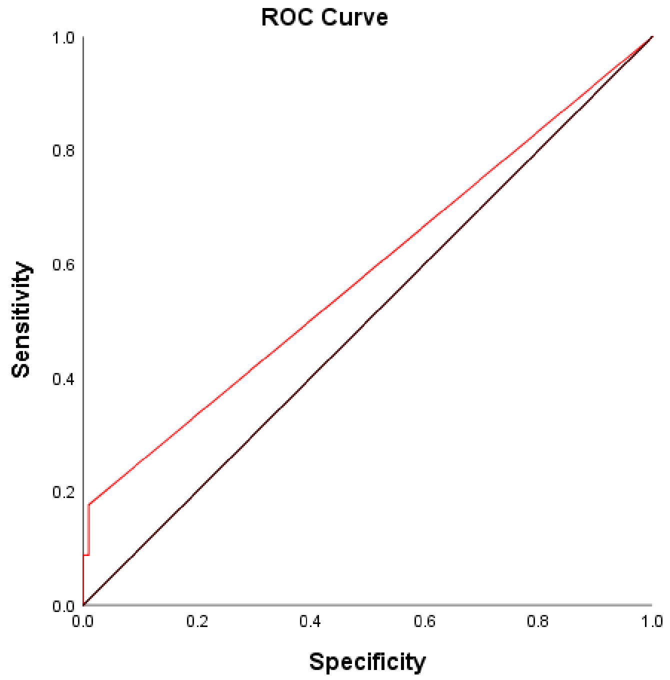

Supplementary Figure S4: Receiver operator characteristic (ROC) curves to assess the discriminatory accuracy of *Fn* DNA detection by qPCR.
